# An unusual case of extensive truncal cutaneous larva migrans in a Cameroonian baby: a case report

**DOI:** 10.1186/s13256-018-1792-y

**Published:** 2018-09-20

**Authors:** Frank-Leonel Tianyi, Valirie Ndip Agbor, Benjamin Momo Kadia, Christian Akem Dimala

**Affiliations:** 1 Mayo-Darlé Sub-divisional Hospital, Banyo, Adamawa Region Cameroon; 2 Ibal sub-Divisional Hospital, Oku, North west Region Cameroon; 3 Foumbot District Hospital, Foumbot, Cameroon; 4 Grace Community Health and Development Association, Kumba, Cameroon; 5 Orthopaedics Department, Southend University Hospital, Essex, UK; 6 Health and Human Development (2HD) Research Network, Douala, Cameroon

**Keywords:** Cutaneous larva migrans, Poor rural settings, Serpentine skin lesions

## Abstract

**Background:**

Cutaneous larva migrans is a neglected zoonotic helminthic disease which is paradoxically underreported in low-income and middle-income countries from where a majority of the cases emanate. It presents as migratory, raised, erythematous serpentine lesions, responsive to treatment with antihelminthics. It is common in children, but rare in babies. We report an unusual case of cutaneous larva migrans in a Cameroonian baby.

**Case presentation:**

We report the case of a 9-month-old Cameroonian baby girl, whose mother had the habit of drying the child’s clothes on the grass in her courtyard. The child was brought to our hospital after she developed itchy, snake-like, slowly progressing lesions on her abdomen and chest. An examination revealed multiple raised serpentine and erythematous skin lesions consistent with cutaneous larva migrans which subsided on antihelminthic and antihistaminic therapy.

**Conclusions:**

Cutaneous larva migrans is an endemic disease with predilection for poor and vulnerable persons. Preventive efforts such as wearing of slippers, usage of drying lines, and deworming of pets are crucial in preventing the occurrence of this disease and should be encouraged.

## Background

Cutaneous larva migrans (CLM) is a neglected zoonotic helminthic disease which results from the cutaneous penetration of larvae of animal parasites [1, 2]. It is a major public health problem, especially in developing countries of the tropics and sub-tropics [1–3]. It is one of the most commonly encountered tropical diseases [4]. However, very few cases are reported in low-income countries where they mostly occur. Paradoxically, most reports are on immigrants returning from endemic areas [5]. The lesions from CLM are typically serpentine and could be a source of stigma and concern among parents and caregivers of affected individuals [2]. This is common in poor rural communities where high rates of illiteracy and superstition cause people to associate some skin conditions with witchcraft [6].

We present the first documented case of CLM in a Cameroonian baby.

## Case presentation

A 9-month-old baby girl from the Adamawa Region of Cameroon was brought to the out-patient department of our hospital by her mother for a reddish, “snake-like” rash on the child’s abdomen that appeared 3 days prior to consultation. The mother suspected the lesions were pruritic because her child was irritable and seemed restless during sleep hours. She reported that the lesions increased in length by approximately 2 cm each day, and they had gotten longer since she first noticed them 3 days prior to consultation. The child had no fever, cough, or other systemic symptoms. They had no pet dogs or cats but our patient’s mother reported that stray dogs usually visit their courtyard. Even though the mother did not allow her children to play in the dirt, she admitted to drying her children’s clothes on the grass in the courtyard. Our patient’s twin sister was symptomless.

**Fig. 1:**
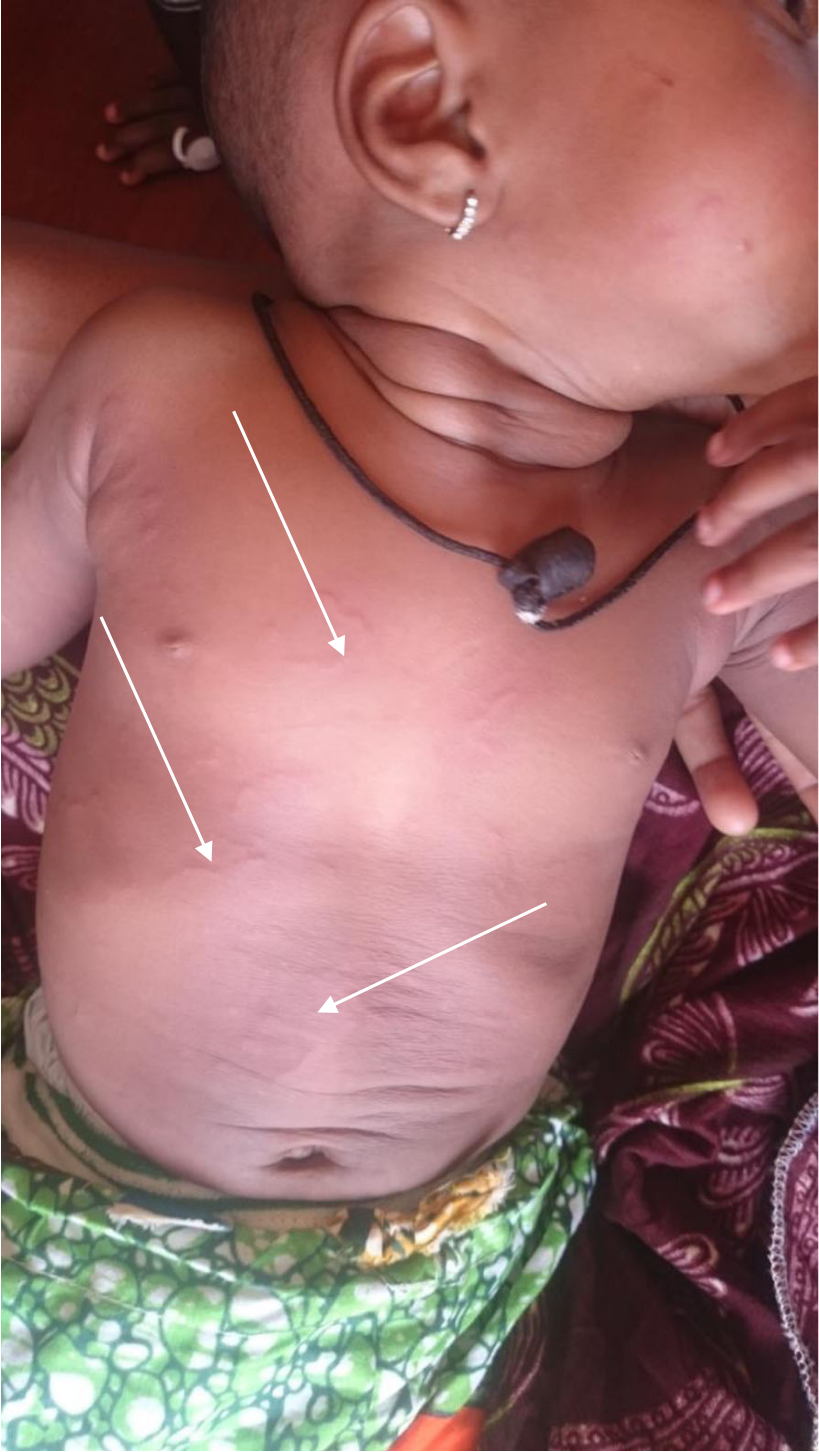
Serpiginous, raised, erythematous skin lesions (*white arrows*)

On physical examination, the child was conscious, calm, and in no form of distress. She had a temperature of 37.4 °C, pulse rate of 92 beats per minute, respiratory rate of 24 breaths per minute, and weighed 9 kg. An examination of her skin revealed multiple erythematous, raised, and “thin” serpiginous lesions of varying lengths over her trunk and extending to the proximal portions of her arms (Fig. 1). The lesions did not appear to increase in length throughout the examination.

A diagnosis of CLM was made and she was placed on albendazole syrup (15 mg/kg per day) for 3 consecutive days and chlorpheniramine syrup 1 mg/ml for 3 days. A follow-up visit 3 days later was marked by absence of irritability but the persistence of a few serpiginous lesions. She was prescribed topical ivermectin cream with a total resolution of the lesions at follow-up, 1 week later.

## Discussion

Hookworm-related CLM (HrCLM) is the commonest cause of creeping eruptions [5, 7]. It is caused by the larvae of dog or cat hookworms such as *Ancylostoma caninum* and *Ancylostoma braziliense* [8]. Even though the global burden of CLM is unknown, a prevalence as high as 18.2% has been reported in Brazil [9]. The situation of the infection in Sub-Saharan Africa (SSA), especially in Cameroon, is still obscure. This is evidenced by the fact that a MEDLINE search via PubMed revealed only five reports on CLM from SSA within a period of 30 years [5, 10–13]. Although there is no racial predilection for CLM, CLM is endemic in Caribbean islands, Africa, South America, South East Asia, and Southeastern USA [1, 14]. Warm temperatures between 23 °C and 30 °C, the presence of loose humid soil, shady areas, and proper aeration all favor larval growth. The degree of contamination and the duration of contact with the soil also influence the occurrence of the disease [15]. This condition is common in childhood, but very rare in infancy, evidenced by a MEDLINE search via PubMed which revealed only four worldwide reports on CLM in infancy from inception [8, 16–18]. A lack of data collection and underreporting of cases of CLM results in an underestimation of the burden of this condition in this part of the continent. This is more of a concern in babies due to their limited range of mobility compared to older children. Hence, they most often do not get infested in the same way. More research on the occurrence of this disease in this age group is needed to explore different means of infestation and other baby-specific information regarding the disease. This will go a long way to help inform stakeholders and policy-makers to implement strategies for disease prevention and eradication.

After being passed in animal (the definitive host) feces, the eggs hatch in warm and moist soil or sand, releasing the infective larvae [5, 19]. The larvae penetrate the human’s (the accidental host) skin on direct contact and secrete proteases and hyaluronidases to ease their migration and penetration through the epidermis [8, 20]. However, the larvae of species such as *Ancylostoma braziliense* are incapable of attaining the dermis as they lack specific collagenases [9, 21]; hence, they wander in the epidermis, producing the pathognomonic serpiginous tracks [1, 22].

The tracks are commonly found in parts of the body which are accidentally exposed to contaminated soil such as the feet, hands, and buttocks [2, 4, 19]. Less common sites include the anterior abdominal wall, breasts, chest, and the penile shaft [8, 23]. In babies, the lesions commonly occur on the buttocks, perineum, and lower abdominal region, given that they often sit, crawl, and play on dirty soil [8, 16]. The occurrence of CLM on the trunk of our patient is atypical. A few cases of atypical presentations (scalp, penile shaft, and breasts) of CLM have been reported in the literature [8, 23, 24]. Also, the extensive nature of the lesions, in addition to their location, supports the classification of our case as an unusual presentation. Our patient’s mother was a stay-at-home mum, and she declared that she never left her children unattended and did not allow them to play in the dirt. However, she admitted to drying the children’s clothes on the grass in the courtyard, placing them at a high risk of contamination from dog and cat feces.

Even though the symptoms of CLM can take weeks to arise [3], an intense pruritus usually 10–15 days after larval penetration is the main presentation [3, 4, 8]. The intense pruritus is due to an intense inflammatory reaction to the hyaluronidases and other enzymes secreted by the larvae to facilitate the penetration of the dermis in a patient with no history of exposure to larvae [15]. In babies, this can be non-specific and manifest as irritability, interfering with sleep [21], as was the case in our patient. The pruritus is followed by a 2–4 mm wide erythematous, elevated, serpiginous track which migrates at a rate of 2 mm to 2 cm per day [3, 19]. These tracks are sometimes multiple and pronounced, and could be a source of stigma as the parents, caregivers, or patients bearing them are usually considered unhygienic [2]. Worse still, in rural communities with low literacy rates and high levels of superstition, these tracks could be associated with witchcraft, resulting in adverse social outcomes for afflicted individuals [25]. As twins are associated with snakes and witchcraft in this community, our patient’s grandmother initially suggested the child be taken to a witch doctor when she saw the “snake-like” lesion. This could cause a delay in effective treatment with the risk of complications such as bacterial superinfection, vesiculobullous lesions, or folliculitis [3, 5, 26]. The children could also be exposed to adverse rituals such as scarification to get rid of the lesions.

The diagnosis is mainly clinical and requires a high degree of suspicion. There are limited resources for investigations and the diagnosis relies on a history of exposure and classic signs and symptoms [1, 2]. Skin biopsy is difficult given that the location of the migrating larva cannot be predicted by the track [3]. A major differential diagnosis is larva currens, which is a fast-moving serpiginous eruption due to skin penetration by larvae of *Strongyloides stercoralis*. In contrast to CLM, the rate of migration is faster at ≥ 5 cm/hour (compared to 1–2 cm per day in CLM). Other differential diagnoses include cercarial dermatitis, contact dermatitis, scabies, and migratory myiasis [8].

The disease is self-limiting, with the larvae dying after 5–6 weeks in the human host [2, 3]. The management is mostly symptomatic with topical and orally administered antihistamines. Orally administered albendazole and ivermectin are curative [2].

Ivermectin is the first line of treatment with single doses of 200 mcg/kg providing 81–100% cure rates [25, 26]. It is well tolerated with little or no side effects. Its safety and efficacy are not established for babies and children weighing less than 15 kg. A single dose of albendazole gives cure rates of 46–100% [25]. It has the advantage of being tolerated in babies over 6 months of age and so could be used in our case [25]. Topical preparations have limited value for multiple lesions or for complications such as hookworm folliculitis [25]. Topical ivermectin is recognized in the management of scabies and rosacea [27, 28]. There is limited evidence on the use of topical ivermectin in the management of CLM. Existing reports are conflicting with some authors finding ivermectin ineffective and others finding it useful in the treatment of CLM [29, 30]. Studies with more robust methodologies need to be carried out to better understand the role of topical ivermectin in the management of CLM.

Complications of CLM include secondary bacterial infection, allergic reactions, and Loeffler’s syndrome [31]. Secondary bacterial infection is the most common complication, occurring in up to 8% of cases [31]. It is facilitated by scratching of the pruritic lesions with a resulting superinfection of the overlying skin. This further complicates the clinical picture, leading to a marked delay in diagnosis and effective treatment [32]. Hence, the initial management should be aimed at reducing the pruritus, eradicating the parasite, and preventing secondary bacterial infection. A worsening of the irritability and the appearance of pustules should highlight to clinicians the possibility of a superinfection and the need to initiate antibiotherapy.

Preventive efforts include: encouraging the use of lines to dry clothes instead of drying clothes on the grass or ground, encouraging children in rural areas to constantly put on shoes or slippers to prevent direct contact with contaminated soil, and deworming of pets for disease eradication [2, 3, 22].

## Conclusions

CLM is a neglected zoonotic disease common among poor and vulnerable persons in rural areas. It is likely that the burden of this disease in Cameroon is underestimated due to underreporting of cases. We recommend observational studies be conducted over the national territory to establish the prevalence and/or incidence of this disease, with a focus on babies to describe disease specificities in this population. Efforts such as usage of drying lines, wearing slippers, and deworming of pets are crucial in preventing the occurrence of this disease and should be encouraged.

## Abbreviations

CLM: Cutaneous larva migrans
HrCLM: Hookworm-related cutaneous larva migrans
SSA: Sub-Saharan Africa
